# Supplementary material for: The interactome of intact mitochondria by cross-linking mass spectrometry provides evidence for coexisting respiratory supercomplexes
Source: Mol Cell Proteomics. 2017 Dec 8;17(2):216–32. doi: 10.1074/mcp.RA117.000470 (PMC5795388; doi:10.1074/mcp.RA117.000470)
Supplement: Supplemental Data [file supp_RA117.000470_133922_0_supp_23978_fzffwf.zip › spectra_annotation/mito_spectra_annotation/62-1-5-1-13-1.pdf]

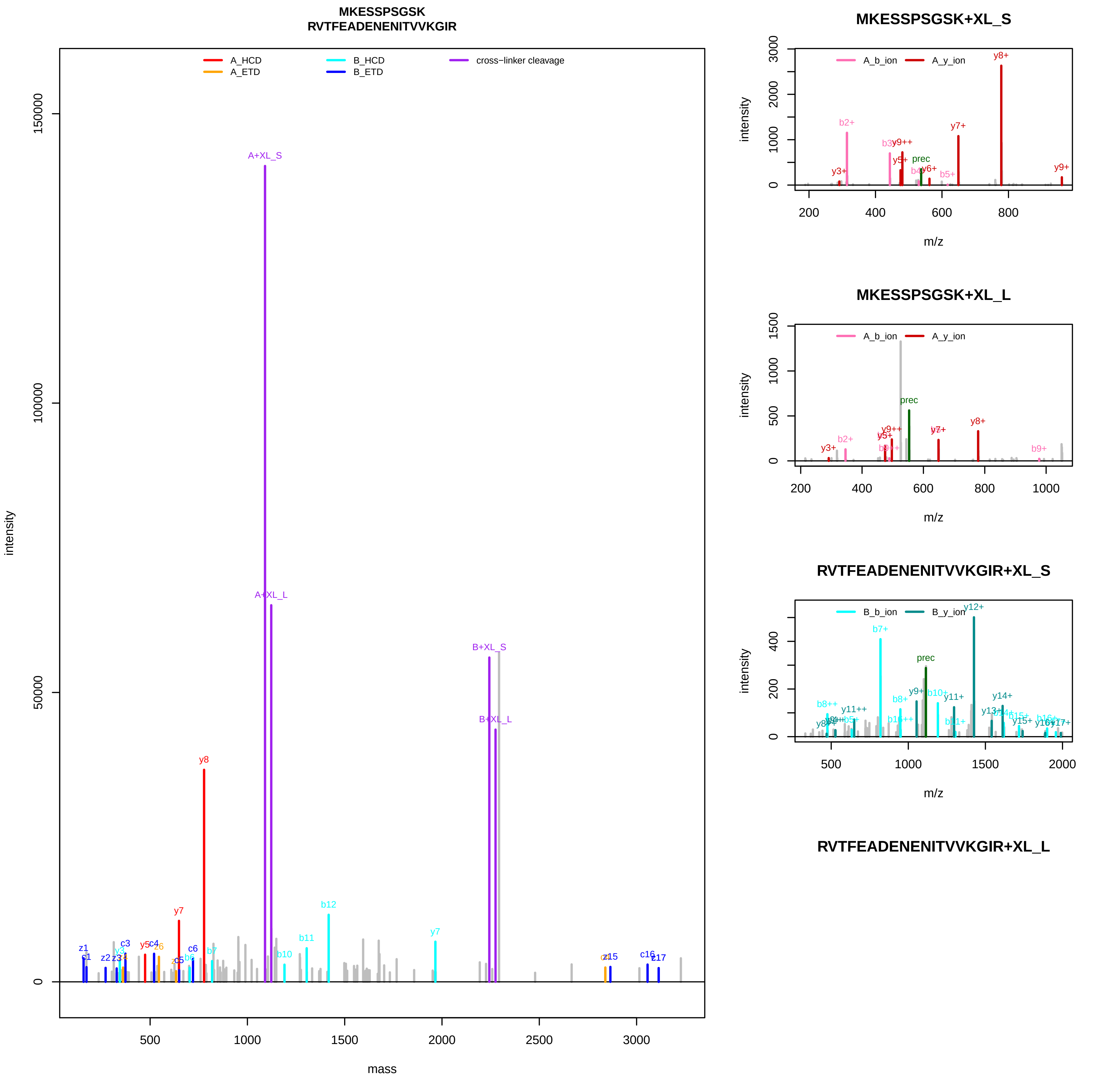
MKESSPSGSK
RVTFEADENENITVVKGIR
A_HCD
A_ETD
B_HCD
B_ETD
cross−linker cleavage
A+XL_S
A+XL_L
B+XL_S
B+XL_L
y8
y7
b12
b11
b10
y7
intensity
mass
0
50000
100000
150000
500
1000
1500
2000
2500
3000
MKESSPSGSK+XL_S
A_b_ion
A_y_ion
y8+
b2+
y7+
b3+
y9++
y5+
prec
y6+
y3+
b5+
y9+
m/z
MKESSPSGSK+XL_L
A_b_ion
A_y_ion
prec
y8+
b2+
y3+
b9+
RVTFEADENENITVVKGIR+XL_S
B_b_ion
B_y_ion
y12+
b7+
prec
y14+
y11+
b10+
y9+
b8+
b8++
y11++
RVTFEADENENITVVKGIR+XL_L
